# Efficacy of immune checkpoint inhibitors in non-small cell lung cancer: A systematic review and meta-analysis

**DOI:** 10.3389/fonc.2022.955440

**Published:** 2022-08-16

**Authors:** Fang Yang, Yucai Wang, Lin Tang, Aaron Scott Mansfield, Alex A. Adjei, Konstantinos Leventakos, Narjust Duma, Jia Wei, Lifeng Wang, Baorui Liu, Julian R. Molina

**Affiliations:** ^1^ The Comprehensive Cancer Center of Nanjing Drum Tower Hospital, The Affiliated Hospital of Nanjing University Medical School & Clinical Cancer Institute of Nanjing University, Nanjing, China; ^2^ Division of Hematology, Mayo Clinic, Rochester, MN, United States; ^3^ Department of Rheumatology and Immunology, The Affiliated Drum Tower Hospital of Nanjing University Medical School, Nanjing, China; ^4^ Division of Medical Oncology, Mayo Clinic, Rochester, MN, United States; ^5^ Lowe Center For Thoracic Oncology, Dana-Farber Cancer Institute, Boston, MA, United States

**Keywords:** non-small cell lung cancer, immune checkpoint inhibitor, efficacy, survival, meta-analysis

## Abstract

**Background:**

Immune checkpoint inhibitors (ICIs) have demonstrated remarkable efficacy in non-small cell lung cancer (NSCLC). However, only a minority of NSCLC patients benefit from ICIs, and whether the magnitude of benefit is specific factor-dependent remains unclear. We performed a systematic review to improve our understanding of clinicopathologic and biomolecular features associated with improved survival upon treatment with ICIs for NSCLC.

**Methods:**

We searched PubMed, Web of Science, Embase, and Scopus from database inception to August 31, 2021, for randomized controlled trials (RCTs) comparing overall survival (OS) in NSCLC treated with ICIs vs control therapies. We calculated the pooled OS hazard ratio (HR) and 95% CI in subgroups using a random-effects model, and assessed the heterogeneity between the paired estimates using an interaction test.

**Results:**

A total of 23 RCTs involving 15,829 patients were included. We found that wild-type EGFR, high PD-L1 expression, and high bTMB were associated with a significant OS benefit from ICIs, but not mutant EGFR, low PD-L1 expression, and low bTMB. The differences of OS benefit between wild-type and mutant EGFR (HR=1.53, 95%CI 1.13-2.08), high and low PD-L1 (HR=1.35; 95%CI 1.14-1.61), high and low bTMB (HR=1.71; 95%CI 1.17-2.52) were statistically significant. OS benefit was found in all subgroups regardless of sex, age, ECOG PS, histology, smoking history, baseline brain metastasis, race, and region, and the interaction test demonstrated no significant difference of the OS benefit between these opposed subgroups (e.g. male vs female).

**Conclusions:**

Wild-type EGFR, high PD-L1 expression, and high bTMB are associated with a greater magnitude of efficacy from ICIs vs control therapies in NSCLC. However, the administration of ICIs should not be restricted to other clinicopathological factors (sex, smoking history, race, etc.).

## Introduction

Immune checkpoint inhibitors (ICIs), including inhibitors of programmed cell death 1 (PD-1), programmed cell death ligand 1 (PD-L1), or cytotoxic T-lymphocyte protein 4 (CTLA-4), have dramatically changed the NSCLC treatment landscape over the past decade. Based on results from multiple global clinical trials, ICIs have been one of the standard first-line NSCLC treatments, as monotherapy or combined therapy (1–6). However, only a minority of NSCLC patients benefit from ICIs (7), and whether the magnitude of benefit is specific factor-dependent remains unclear. Given the adverse effects (8) and cost of ICIs, and the lack of access to the drug in low-to-middle income countries, it is crucial to identify subpopulations who can derive a larger relative benefit from immunotherapy.

Anti-PD-1/PD-L1 therapy works by blocking the PD-1/PD-L1 pathway, suggesting that high PD-L1 expression could be a reasonable biomarker predicting the efficacy. However, some PD-L1-negative patients also respond to anti-PD-1/PD-L1 therapy (9). Moreover, currently there is no unified standard for defining PD-L1 positivity. Whether there are differences in the relative benefit from ICIs over control therapies remains unclear when using different positive PD-L1 standards.

Sex, age and Eastern Cooperative Oncology Group (ECOG) performance status (PS) might correlate with immune response, thus affecting the efficacy of ICIs. We have explored the association of these three variables with the relative benefit of ICIs in solid tumors. Stratified analysis showed no difference in the survival advantage of immunotherapy among NSCLC patients grouped by sex, age, and ECOG PS (10). However, other clinicopathological and biomolecular characteristics were not analyzed, and several large clinical trials have published updated results in recent years, warranting an updated analysis.

NSCLC harboring epidermal growth factor receptor (EGFR) mutations or low tumor mutation burden (TMB) showed poor clinical outcomes with immunotherapy (11, 12). Histological type, smoking status and race are known to correlate with NSCLC EGFR mutation rate. NSCLC brain metastases showed an increased TMB and genomic instability in comparison with primary NSCLC (13). Therefore, these clinical features mentioned above may also be associated with therapeutic benefit of ICIs.

Given the lack of data on the above issues, we conducted a comprehensive meta-analysis to examine the potential association of the common clinicopathological and biomolecular features with relative advantages of immunotherapy in NSCLC.

## Methods

The study was registered with PROSPERO (CRD42019123892), an international prospective register of systematic reviews, and performed following the Preferred Reporting Items for Systematic Reviews and Meta-analyses (PRISMA) guidelines (14). The need for institutional review board approval was waived by Drum Tower Hospital because this meta-analysis utilized publicly available data.

### Study selection and data extraction

We performed a systematic literature search using PubMed, Web of Science, Embase, and Scopus for phase 2/3 randomized controlled trials (RCTs) from database inception to August 31, 2021. Two investigators (FY and YW) independently searched the databases. Any disagreement was resolved by discussion and consensus. We performed full-text reviews if abstracts were insufficient for determining if the studies met the inclusion criteria. We set the search criteria to include all the PD-1 inhibitors (nivolumab, pembrolizumab, cemiplimab, toripalimab, sintilimab, camrelizumab, tislelizumab, penpulimab, zimberelimab), PD-L1 inhibitors (durvalumab, atezolizumab, avelumab, sugemalimab), and CTLA-4 inhibitors (ipilimumab, tremelimumab) in NSCLC. The references of the included studies were also reviewed for potential additional publications.

Eligible studies met all of the following requirements (1): RCTs assessing PD-1/PD-L1 or CTLA-4 inhibitors for treatment of NSCLC (2); ICIs as monotherapy or part of combination therapy in the intervention arm, and control therapy without ICIs in the control arm (3); data available for hazard ratio (HR) for overall survival (OS) in subgroups defined by clinicopathological and/or biomolecular characteristics; and (4) published in English. If subgroup data of a study were reported in more than one publication, the most updated or comprehensive data were included in this analysis. Outcome data was extracted, including HR and 95% confidence interval (CI) stratified by sex, age, ECOG PS, histology, smoking status, baseline brain metastases, EGFR mutation, PD-L1 expression, TMB, race, and regions.

### Statistical analysis

We calculated the pooled HRs of death in each of the paired subgroups (e.g., male vs female) using the random-effects models to determine whether any subgroup of patients benefited from ICI vs control therapy.

We calculated a study-specific interaction HR (95%CI) in each study based on the reported HRs (95%CIs) in paired subgroups and then combined the study-specific interaction HRs across trials, using a random-effects model, to generate a P value for heterogeneity (P_heterogeneity_) as described previously (10, 15, 16). A P_heterogeneity_<0.05 indicated that the magnitude of OS benefit from ICI vs control therapy was different between the paired subgroups (e.g., male vs female). The between-study heterogeneity was assessed by the Q test and quantified by I^2^ values. I^2^ value <=25% corresponds to a low heterogeneity (17).

All analyses were conducted using Comprehensive Meta-Analysis, version 2. All reported P values are two-sided, and a P<0.05 was considered statistically significant.

## Results

### Literature search results and characteristics of identified trials

**Figure 1:**
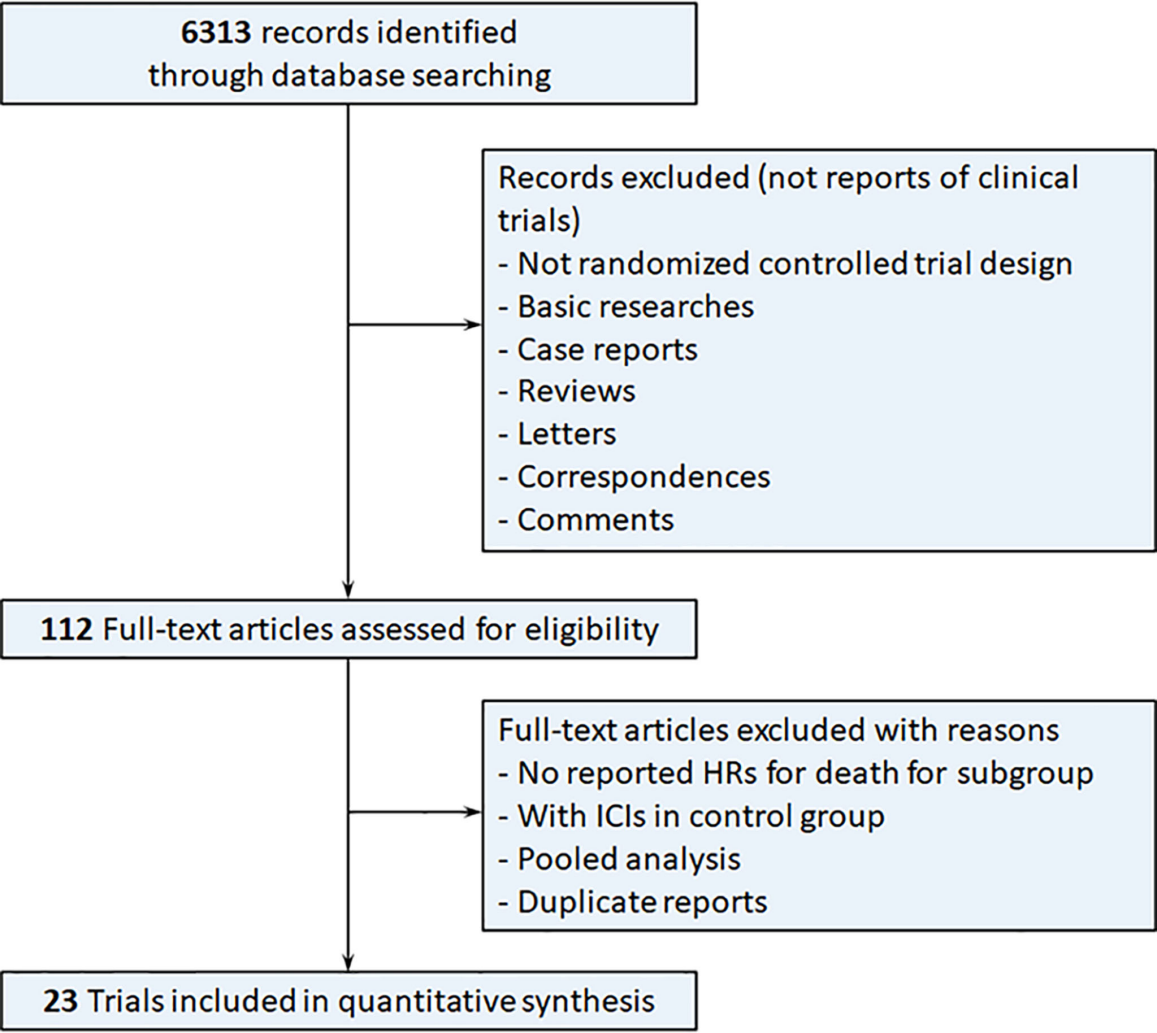
Flow diagram of the study selection process. HRs, hazard ratios; ICIs, immune checkpoint inhibitors.

The systematic search yielded 6313 results, of which 112 were reviewed in full. Finally, 23 RTCs involving 15829 patients, published from 2015 to 2021, satisfied our inclusion criteria and were included in the meta-analysis (**Figure 1**) (2, 3, 5, 6, 18–48). Characteristics of the included trials are summarized in **Table 1**. Most of the trials were phase 3. We found 15 trials conducted for first line, 8 for subsequent lines, 11 trials with anti-PD-1 inhibitors, 10 trials with anti-PD-L1 inhibitors, 1 trial with anti-CTLA-4 inhibitor, and 2 trials with combined ICIs, 14 trials compared immunotherapy alone to control therapy, and 9 trials compared combination of immunotherapy and chemotherapy to control therapy.

**Table 1:** List of the studies included in this meta-analysis.

Trial name	Source	Phase	NCT No.	Total patient No.	Line of therapy	Treatment arms
CheckMate 017	Brahmer et al. (2015) (18)	3	NCT01642004	272	>1	nivolumab vs chemotherapy
CheckMate 026	Carbone et al. (2017) (19)	3	NCT02041533	541	1	nivolumab vs chemotherapy
CheckMate 057	Borghaei et al. (2015) (20)	3	NCT01673867	582	>1	nivolumab vs chemotherapy
CheckMate 078	Lu et al. (2021) (21)	3	NCT02613507	504	>1	nivolumab vs chemotherapy
CheckMate 9LA	Paz-Ares et al. (2021) (22)	3	NCT03215706	719	1	nivolumab+ipilimuma+chemotherapy vs chemotherapy
EMPOWER-Lung 1	Sezer et al. (2021) (6)	3	NCT03088540	710	1	cemiplimab vs chemotherapy
IMpower110	Herbst et al. (2020) (5) Jassem et al. (2021) (23)	3	NCT02409342	554	1	atezolizumab vs chemotherapy
IMpower130	West et al. (2019) (3)	3	NCT02367781	679	1	atezolizumab+chemotherapy vs chemotherapy
IMpower131	Jotte et al. (2020) (24)	3	NCT02367794	683	1	A+CnP vs CnP
IMpower132	Nishio et al. (2021) (25)	3	NCT02657434	578	1	APP vs PP
IMpower150	Reck et al. (2019) (26) Socinski et al. (2021) (27)	3	NCT02366143	1202	1	ACP vs BCP ABCP vs BCP
JAVELIN Lung 200	Barlesi et al. (2018) (28) Park et al. (2021) (29)	3	NCT02395172	529	>1	avelumab vs chemotherapy
KEYNOTE-010	Herbst et al. (2016) (30) Herbst et al. (2020) (31) Herbst et al. (2021) (32)	2/3	NCT01905657	1033	>1	pembrolizumab vs chemotherapy
KEYNOTE-024	Reck et al. (2019) (33)	3	NCT02142738	305	1	pembrolizumab vs chemotherapy
KEYNOTE-042	Mok et al. (2019) (2)	3	NCT02220894	1274	1	pembrolizumab vs chemotherapy
KEYNOTE-189	Gandhi et al. (2018) (34) Rodríguez-Abreu et al. (2021) (35)	3	NCT02578680	616	1	pembrolizumab+chemotherapy vs placebo+chemotherapy
KEYNOTE-407	Paz-Ares et al. (2018) (36) Paz-Ares et al. (2020) (37)	3	NCT02775435	559	1	pembrolizumab+chemotherapy vs placebo+chemotherapy
MYSTIC	Rizvi et al. (2020) (38)	3	NCT02453282	1118	1	durvalumab vs chemotherapy durvalumab+tremelimumab vs chemotherapy
NCT01285609	Govindan et al. (2017) (39)	3	NCT01285609	749	1	ipilimumab+chemotherapy vs placebo+chemotherapy
OAK	Rittmeyer et al. (2017) (40) Fehrenbacher et al. (2018) (41) Hida et al. (2018) (42) Gadgeel et al. (2019) (43)	3	NCT02008227	1225	>1	atezolizumab vs chemotherapy
ORIENT-11	Yang et al. (2021) (44)	3	NCT03607539	397	1	sintilimab+chemotherapy vs placebo+chemotherapy
PACIFIC	Antonia et al. (2018) (45) Paz-Ares et al. (2020) (46) Faivre-Finn et al. (2021) (47)	3	NCT02125461	713	>1	durvalumab vs placebo
POPLAR	Fehrenbacher et al. (2016) (48)	2	NCT01903993	287	>1	atezolizumab vs chemotherapy

A+CP, atezolizumab+carboplatin+paclitaxel; A+CnP, atezolizumab+carboplatin+nab-paclitaxel; APP, atezolizumab+carboplatin/cisplatin+pemetrexed; PP, carboplatin/cisplatin + pemetrexed; ABCP, atezolizumab+bevacizumab+carboplatin+paclitaxel; BCP, bevacizumab+carboplatin+paclitaxel.

### Associations of clinicopathological and biomolecular characteristics with OS

Firstly, we analyzed the associations between clinicopathological characteristics and the relative OS benefit of immunotherapy over control therapy (**Table 2**). Patients with NSCLC derived an OS benefit from ICIs regardless of sex, age, ECOG PS, histological type, smoking status, and baseline brain metastasis status, and the magnitude of OS benefit was not significantly different between paired subgroups (all P_heterogeneity_>0.05) (**Table 2**). Patients with wild-type EGFR significantly benefited from immunotherapy (HR=0.71, 95%CI: 0.64-0.78), but patients with mutant EGFR did not (HR=1.08, 95%CI: 0.81-1.44) (**Table 2**). The interaction test confirmed the magnitude of OS benefit was significantly different between the two groups (P_heterogeneity_=0.006) (**Table 2**).

**Table 2:** Differences in efficacy of IO vs control therapies by subgroups.

Variable	Studies No.	Patients No.	Pooled HR (95% CI)	P	P_heterogeneity_	Between-study heterogeneity		
						Q	P	I^2^, %
Sex	20				0.715			
Male		9232	0.76 (0.72-0.81)	<0.001		26.67	0.145	25.02
Female		4459	0.78 (0.69-0.87)	<0.001		40.72	0.004	50.89
Age	20				0.270			
<65		7162	0.75 (0.69-0.81)	<0.001		38.03	0.009	47.42
>=65		5918	0.79 (0.74-0.85)	<0.001		21.69	0.357	7.81
ECOG PS	20				0.442			
0		4583	0.76 (0.69-0.83)	<0.001		24.92	0.204	19.75
1		8563	0.76 (0.71-0.82)	<0.001		36.00	0.011	47.22
Histology	12				0.396			
Squamous		2618	0.74 (0.67-0.81)	<0.001		7.39	0.766	<0.001
Nonsquamous		5364	0.78 (0.70-0.87)	<0.001		23.16	0.017	52.50
Smoking	17				0.313			
Never		1831	0.82 (0.70-0.96)	0.014		29.20	0.033	41.78
Former/current		9259	0.76 (0.71-0.81)	<0.001		29.94	0.027	43.22
Brain metastasis	7				0.110			
Yes		585	0.55 (0.41-0.73)	<0.001		9.77	0.135	38.59
No		3969	0.70 (0.63-0.78)	<0.001		8.98	0.175	33.19
*EGFR*	4				0.006			
Mutant		325	1.08 (0.81-1.44)	0.616		0.72	0.868	<0.001
WT		2615	0.71 (0.64-0.78)	<0.001		1.69	0.640	<0.001

IO, immunotherapy; ECOG PS, Eastern Cooperative Oncology Group performance status; EGFR, epidermal growth factor receptor; WT, wild type; HR, hazard ratio; CI, confidence interval.

Secondly, we analyzed the associations of OS benefit from ICIs with PD-L1 expression and bTMB. For PD-L1 expression, different cutoffs of tumor proportion score (TPS), tumor cell (TC), or immune cell (IC) were used in various studies. Patients with TPS/TC>=50% significantly benefited from immunotherapy (HR=0.66, 95%CI: 0.59-0.74), but patients with TPS/TC<50% did not (HR=0.88, 95%CI: 0.74-1.06). The interaction test confirmed the magnitude of OS benefit was significantly different between the two groups (P_heterogeneity_=0.001) (**Table 3**). Patients with TPS/TC>=1% or <1% both benefited from immunotherapy, with no statistical difference between the magnitude of OS benefit (P_heterogeneity_=0.521) (**Table 3**). Patients with TC/IC=1/2/3 significantly benefited from immunotherapy (HR=0.73, 95%CI: 0.64-0.82), but patients with TC/IC=0 did not (HR=0.90, 95%CI: 0.79-1.02). The magnitude of OS benefit was significantly different between the two groups (P_heterogeneity_=0.020) (**Table 3**).

**Table 3:** Differences in efficacy of IO vs control therapies by PD-L1 expression and bTMB.

Variable	Studies No.	Total patients No.	Subgroup	Patients No.	HR (95% CI)	P	P_heterogeneity_	Between-study heterogeneity		
								Q	P	I^2^, %
PD-L1										
TPS/TC<50% vs TPS/TC>=50%	5	4108	TPS/TC<50%	2245	0.88 (0.74-1.06)	0.186	0.001	16.75	0.005	70.16
			TPS/TC>=50%	1863	0.66 (0.59-0.74)	<0.001		4.32	0.505	<0.001
TPS/TC<1% vs TPS/TC>=1%	8	4444	TPS/TC<1%	1490	0.75 (0.62-0.90)	0.002	0.521	17.95	0.022	55.44
			TPS/TC>=1%	2954	0.71 (0.61-0.82)	<0.001		22.81	0.004	64.92
TC/IC=0 vs TC/IC=1/2/3	3	2548	TC/IC=0	1127	0.90 (0.79-1.02)	0.096	0.020	1.04	0.792	<0.001
			TC/IC=1/2/3	1421	0.73 (0.64-0.82)	<0.001		1.60	0.660	<0.001
bTMB										
<20mut/Mb vs >=20mut/Mb	2	1198	<20mut/Mb	931	0.98 (0.83-1.16)	0.856	0.006	3.25	0.197	38.51
			>=20mut/Mb	267	0.59 (0.46-0.76)	<0.001		2.03	0.363	1.21
<16mut/Mb vs >=16mut/Mb	2	1453	<16mut/Mb	945	1.01 (0.84-1.22)	0.876	0.013	3.42	0.181	41.58
			>=16mut/Mb	508	0.67 (0.54-0.84)	<0.001		2.35	0.310	14.70

IO, immunotherapy; PD-L1, programmed cell death 1 ligand 1; bTMB, blood tumor mutation burden; TPS, tumor proportion score; TC, tumor cell; IC, immune cell; HR, hazard ratio; CI, confidence interval.

We found that patients with higher bTMB significantly benefited from ICI (>=20mut/Mb: HR=0.59, 95%CI: 0.46-0.76; >=16mut/Mb: HR=0.67, 95%CI: 0.54-0.84), but patients with lower bTMB did not (<20mut/Mb; HR=0.98, 95%CI: 0.83-1.16; <16mut/Mb: HR=1.01, 95%CI: 0.84-1.22) (**Table 3**). The interaction test confirmed the magnitude of OS benefit was significantly different between high and low bTMB groups (cutoff of 20mut/Mb: P_heterogeneity_=0.006; cutoff of 16mut/Mb: P_heterogeneity_=0.013) (**Table 3**).

Additionally, we analyzed the associations of patient race and region with OS benefit from ICIs (**Table 4**). We found that nearly all patients with NSCLC benefited from ICIs over control therapies regardless of race and region. Black/African American patients were under-represented in the studies, with 45 patients included in 4 studies, accounting for only 1.8%-7.0% of the total population size. The OS benefit they derived from ICIs appeared to be statistically insignificant, likely due to a very small sample size. The interaction test demonstrated that there was no significant difference in the magnitude of OS benefit between paired subgroups (all P_heterogeneity_>0.05) (**Table 4**).

**Table 4:** Differences in efficacy of IO vs control therapies by race and region.

Variable	Studies No.	Total patients No.	Subgroup	Patients No.	HR (95% CI)	P	P_heterogeneity_	Between-study heterogeneity		
								Q	P	I^2^, %
Race										
Asian vs Non-Asian	8	4779	Asian	1063	0.82 (0.70-0.96)	0.012	0.884	6.35	0.608	<0.001
			Non-Asian	3716	0.84 (0.78-0.90)	<0.001		5.51	0.702	<0.001
White vs Non-White	8	5458	White	4244	0.81 (0.74-0.88)	<0.001	0.586	12.03	0.150	33.52
			Non-White	1214	0.84 (0.72-0.97)	0.016		5.75	0.675	<0.001
Asian vs White	7	4454	Asian	957	0.84 (0.71-0.99)	0.035	0.990	5.75	0.570	<0.001
			White	3497	0.84 (0.78-0.90)	<0.001		5.38	0.613	<0.001
Asian vs Black/African American	4	639	Asian	594	0.89 (0.73-1.08)	0.247	0.840	3.07	0.547	<0.001
			Black/African American	45	0.88 (0.43-1.83)	0.736		0.45	0.978	<0.001
White vs Black/African American	4	2483	White	2438	0.85 (0.78-0.93)	<0.001	0.940	1.74	0.784	<0.001
			Black/African American	45	0.88 (0.43-1.83)	0.736		0.45	0.978	<0.001
Region										
Asia vs Non-Asia	9	5625	Asia	1376	0.79 (0.67-0.92)	0.003	0.943	8.93	0.348	10.41
			Non-Asia	4249	0.75 (0.69-0.81)	<0.001		7.99	0.434	<0.001
Asia vs Europe	5	2420	Asia	670	0.86 (0.70-1.05)	0.136	0.730	3.39	0.496	<0.001
			Europe	1750	0.77 (0.68-0.88)	<0.001		3.85	0.427	<0.001
Asia vs America	4	1307	Asia	593	0.84 (0.68-1.03)	0.096	0.174	2.47	0.480	<0.001
			America	714	0.66 (0.48-0.91)	0.011		7.40	0.060	59.43
America vs Europe	6	2636	America	1015	0.62 (0.50-0.77)	<0.001	0.053	9.11	0.105	45.12
			Europe	1621	0.78 (0.67-0.90)	0.001		6.71	0.243	25.52

IO, immunotherapy; HR, hazard ratio; CI, confidence interval.

### Subgroup analyses

We performed subgroup analyses to investigate whether immunotherapy settings affected the relative OS benefit in patients with different were sex, age, ECOG PS, histological type, and smoking status. Specifically, we analyzed the data according to line of therapy (first line or subsequent lines), immunotherapy agent (PD-1 inhibitor or PD-L1 inhibitor), and immunotherapy intervention (immunotherapy alone or chemo-immunotherapy). We found that regardless of the line of treatment, immunotherapy agent, and immunotherapy intervention, the magnitude of OS benefit from ICIs was similar for male vs female, <65 vs >=65 years, ECOG PS=0 vs ECOG PS>=1, squamous vs nonsquamous, and smokers vs non-smokers (**Supplementary Tables 1–5**).

### Between-study heterogeneity analyses

Between-study heterogeneity was found for female patients (Q=40.72, P=0.004, I²=50.89%), <65 years (Q=38.03, P=0.009, I²=47.42%), ECOG PS>=1 (Q=36.00, P=0.011, I²=47.22%), nonsquamous (Q=23.16, P=0.017, I²=52.50%), never smokers (Q=29.20, P=0.033, I²=41.78%), former or current smokers (Q=29.94, P=0.027, I²=43.22%), PD-L1 TPS/TC<50% (Q=16.75, P=0.005, I²=70.16%), PD-L1 TPS/TC<1% (Q=17.95, P=0.022, I²=55.44%), and PD-L1 TPS/TC>=1% (Q=22.81, P=0.004, I²=64.92%) (**Tables 2**, **3**).

## Discussions

In this meta-analysis, we found that wild-type EGFR, high PD-L1 expression, and high bTMB were associated with a greater OS benefit from ICI therapy vs control therapies in patients with NSCLC. Clinicopathological features such as sex, age, ECOG PS, histological type, smoking history, or baseline brain metastasis status (previously treated) were not associated with OS benefit from ICI therapy. These results highlight the importance of EGFR, PD-L1 expression and bTMB as predictive markers for NSCLC immunotherapy. Moreover, our study suggests that the use of ICIs in NSCLC should not be restricted to certain factors such as sex, smoking history, race, etc.

A growing body of literature has illustrated sex­based differences in immune responses. Adult females mount stronger innate and adaptive immune responses than males (49), thus could potentially influence the efficacy of immunotherapy. In addition to sex, age and ECOG PS have also been reported to potentially influence the response to ICIs (50, 51). However, we did not find any statistically significant difference in OS benefit from ICIs in patients with different sex, age, or ECOG PS, both in our previous (10) and the current study. Many large-scale clinical trials of immunotherapy were carried out in different races and regions around the world, but there is no research on whether OS benefit from ICI vs control therapy differs by the patient’s race or region. We found that there was no difference in the degree of relative benefit from immunotherapy between races and regions. These findings suggest that ICI immunotherapy is beneficial in NSCLC patients regardless of sex, age, ECOG PS, race and region, and the use of ICIs should not be restricted to any subgroups based on these variables.

Brain metastases are associated with low quality of life and poor survival outcome. The blood-brain barrier extremely limits the therapeutic effectiveness of drugs. A phase 2 trial has demonstrated the positive role of pembrolizumab in treating NSCLC with brain metastases, as the first prospective study of immunotherapy focusing on brain metastasis. The brain metastasis response rate was 29.7% in the PD-L1 TPS≥1% population, with 4 patients achieved complete response and 7 achieved partial response, meeting the prespecified success criteria set for the trial (52). The subgroup analyses from some clinical trials did show that NSCLC patients with brain metastases could benefit from immunotherapies over control therapies (22, 34, 35, 40), whereas some other trials did not (6, 20, 33, 44). The majority of clinical trials with ICIs require that brain metastases be treated by surgical resection or radiation with a subsequent period of stability prior to enrollment. Pooled analyses have suggested that these patients with treated brain metastases still derive significant benefit with ICIs (53, 54). Here, we found NSCLC patients with baseline brain metastases could also benefit from the ICIs, and the magnitude of benefit was similar to that in patients without baseline brain metastases. The results suggest the possibility that ICIs may pass the (likely disrupted) blood-brain barrier and treat brain metastases effectively. However, the number, size, and location of brain metastases may influence the efficacy of ICIs, which needs to be analyzed in future studies.

We demonstrated ICIs resulted in an improved OS compared with controls among EGFR wild-type patients but not among EGFR-mutant patients, consistent with several previous publications (55, 56). Notably, a minority of patients with EGFR-mutant NSCLC could still benefit from ICIs. For example, NSCLC patients with L858 mutations had a similar outcome with ICI treatment compared with those with wild-type EGFR in a large retrospective study (11). Moreover, high PD-L1 expression in patients with EGFR-mutant NSCLC showed a trend toward better outcomes than those with low PD-L1 expression (57). Additionally, add immunotherapy to certain backbone therapies could significantly improve outcomes of NSCLC patients with TKI sensitive EGFR mutations (26). Therefore, NSCLC patients with sensitive EGFR mutations might still benefit from immunotherapy if there is high PD-L1 expression or when used in combination with chemotherapy (58–60). Mutation status in KRAS, TP53, MET, and ROS were associated with PD-L1 expression in patients with lung adenocarcinomas (61, 62). ALK rearrangement status may be associated with response to PD-1/PD-L1 pathway blockade (55). Given the small number of trials, we did not analyze the difference of survival benefit by other genetic mutations.

As the target of anti-PD-L1 antibodies, expression of PD-L1 has become the major initial predictor of benefit from immunotherapy. High tumor PD-L1 expression is correlated with an increased likelihood of response to anti-PD-1/PD-L1 antibodies. In this study, we grouped studies that used the same cutoffs of PD-L1 expression to analyzed the association with OS benefit. Pooled analysis shows that patients with TPS/TC<1% can also benefit from ICIs. The positive result may be partly attributed to some included trials reporting combined ICIs (CTLA4 antibody + PD-1/PD-L1 antibody). A greater OS benefit was observed in those with high PD-L1 levels when dividing subgroups by 50% tumoral PD-L1 expression, but no difference was found when using 1% as the cutoff. This suggested that NSCLC patients with a significantly higher PD-L1 expression may benefit more from ICIs than other groups, but that benefit isn’t restricted to patients with detectable PD-L1 expression. Quantifying PD-L1 expression on immune cells in addition to tumor cells seemed to strengthen the predictive value of PD-L1 for OS benefit, as immune cells expressing PD-L1 also play a key role in regulating antitumor immune response. There are some challenges in establishing PD-L1 expression as a reliable predictive biomarker. First, the attempts to take PD-L1 as a predictor of immunotherapy have yielded variable results using different cutoffs. Second, the antibodies and testing platforms varied, e.g., SP263 (durvamab) and SP142 (atezolizumab) on the Ventana platform, 22C3 (pembrolizumab) and 28-8 (nivolumab) on the DAKO platform, and 73-10 on the Abcam platform (avelumab). It is hard to reach a uniform standard for the detection of PD-L1 expression. The International Association for the Study of Lung Cancer Pathology Committee has made effort to harmonize and standardize testing for PD-L1 by immunohistochemistry (63). Third, PD-L1 expression may have a great temporal and spatial heterogeneity. In one study, the concordance rate for PD-L1 levels was only 67% between paired samples collected more than three months apart (64). In another study, assessment of different fields of view in the same patient sample showed discordant expression at a frequency of 25% (65). We have previously made significant effort to identified the heterogeneity of PDL1 between the tumor microenvironment of paired primary lung cancers and metastatic lesions (66–69). Finally, irresponsiveness and rapid disease progression can be observed in patients with high tumoral PD-L1 expression, while conversely responses can still occur in PD-L1 negative tumors (70). The CheckMate 227 trial showed a similar survival outcome between the high and low PD-L1 expression in NSCLC patient with a high TMB (71). The results indicated that combining biomarkers, such as PD-L1 and TMB, may increase the predictive efficiency.

As described previously, TMB could be a potential biomarker predicting better response to ICIs. Tumors with high TMB are more likely to generate neoantigens for immune recognition and tumor cell killing, thus resulting in stronger antitumor immune responses to PD-1/PD-L1 or CTLA-4 blockade. NCCN guidelines endorsed TMB as a predictor of immunotherapy based on the positive findings in advanced NSCLC from CheckMate 227 (4). CheckMate 227 also assessed the benefit of ICIs for patients with TMB^high^PD-L1^low^, and found that these patients could significantly benefit from ICIs (HR 0.51, 95%CI 0.30–0.87) (4). The findings suggested that combining TMB with PD-L1 can identify more patients who can potentially benefit from ICIs. This need to be verified in a larger sample size. The result in our study was consistent with previous publications, but our analysis is limited by the few trials reporting this analysis. A larger effort is needed before taking TMB as a convenient and affordable tool in clinical practice. The first challenge is the use of variable cutoffs for high TMB among different studies and between blood or tumor tissues (72–75).

Our study has several strengths. First, we performed an exceedingly comprehensive literature search and included most updated data. Second, we included multiple clinicopathological and biomolecular characteristics in the analysis, some of which such as race and region were analyzed for the first time. Third, we performed analyses with various cutoffs for PD-L1 and bTMB as well as different categorization of race and region. Additionally, we performed subgroups analyses to explore whether immunotherapy settings affected the association of ICI OS benefit with clinical variable such as sex, age and ECOG PS.

Our study also has limitations. First, we conducted the analysis based on published study-level data but not on individual patient-level data. Second, statistically significant heterogeneity was found among the studies for some subgroups. Third, the number of studies that were included in the subgroups analyses and in TMB analysis was small. Fourth, clinical trial enrollment criteria limit the range of many variables that are available for our analysis. For example, patients with an ECOG PS of 2 or lower typically are not included in trials, and patients with brain metastasis typically have undergone resection or received radiation. Finally, our results may not be generalizable to all patients and clinician experiences, because we did not include non-English studies and real-world studies.

## Conclusions

This meta-analysis, which included an interaction test, demonstrated that the OS benefit from ICIs did not appear to differ on the basis of patients’ sex, age, ECOG PS, histology, smoking history, baseline brain metastasis, race, and region. These data suggest that the use of ICIs in NSCLC patients should not be restricted by those variables. However, EGFR status, PD-L1 expression, and TMB are important biomarkers that could potentially predict the efficacy of ICIs. The value of using multiple factors in predicting the efficacy of ICIs might be an important future research topic.

## Data availability statement

The original contributions presented in the study are included in the article/**Supplementary Material**. Further inquiries can be directed to the corresponding authors.

## Author contributions

Conception and design: FY, YW, and JM. Collection and assembly of data: FY, YW, and LT. Assessed the eligibilities of feasible studies: FY, YW, and LT. Statistical analysis: FY, YW, and LT. Wrote the first draft of the manuscript: FY and YW. Revised and edited the manuscript: all authors. Final approval of manuscript: all authors.

## Funding

This research was funded by National Natural Science Foundation of China (No. 82002783), Nanjing Outstanding Youth Fund (No. JQX21001), The Double Innovation Talent Program of Jiangsu Province (2019).

## Conflict of Interest

The authors declare that the research was conducted in the absence of any commercial or financial relationships that could be construed as a potential conflict of interest.

## Publisher’s note

All claims expressed in this article are solely those of the authors and do not necessarily represent those of their affiliated organizations, or those of the publisher, the editors and the reviewers. Any product that may be evaluated in this article, or claim that may be made by its manufacturer, is not guaranteed or endorsed by the publisher.
