# Supplementary material for: Efficacy of immune checkpoint inhibitors in non-small cell lung cancer: A systematic review and meta-analysis
Source: Front Oncol. 2022 Aug 16;12:955440. doi: 10.3389/fonc.2022.955440 (PMC9425065; doi:10.3389/fonc.2022.955440)
Supplement: Supplementary file 1 [file DataSheet_1.docx]

Supplementary Material

**Supplementary Table 1.** Differences in efficacy of IO vs control therapies in NSCLC patients by sex.

| **Variable** | **Studies No.** | **Patients No.** | | **Pooled HR (95% CI)** | | **Test for difference** | |
| --- | --- | --- | --- | --- | --- | --- | --- |
|  |  | Male | Female | Male | Female | I^2^, % | P_heterogeneity_ |
| Overall | 20 | 9232 | 4459 | 0.76 (0.72-0.81) | 0.78 (0.69-0.87) | 21.12 | 0.715 |
| Line of therapy | |  |  |  |  |  |  |
| First line | 14 | 6446 | 2891 | 0.77 (0.71-0.83) | 0.79 (0.67-0.92) | 38.05 | 0.734 |
| Subsequent lines | 6 | 2786 | 1568 | 0.74 (0.68-0.81) | 0.75 (0.65-0.87) | <0.01 | 0.940 |
| Agent of immunotherapy | | |  |  |  |  |  |
| PD-1 inhibitor | 10 | 4309 | 1980 | 0.71 (0.64-0.78) | 0.72 (0.58-0.90) | 40.83 | 0.921 |
| PD-L1 inhibitor | 8 | 3784 | 2150 | 0.81 (0.75-0.88) | 0.81 (0.74-0.90) | <0.01 | 0.885 |
| Intervention | |  |  |  |  |  |  |
| Immunotherapy alone | 12 | 5335 | 2571 | 0.74 (0.68-0.80) | 0.80 (0.72-0.89) | <0.01 | 0.357 |
| Chemo-immunotherapy | 8 | 3897 | 1888 | 0.79 (0.73-0.86) | 0.74 (0.59-0.91) | 53.18 | 0.658 |

IO: immunotherapy; NSCLC: non-small cell lung cancer; PD-1: programmed cell death 1; PD-L1: programmed cell death ligand 1; HR: hazard ratio; CI: confidence interval.

**Supplementary Table 2.** Differences in efficacy of IO vs control therapies in NSCLC patients by age.

| **Variable** | **Studies No.** | **Patients No.** | | **Pooled HR (95% CI)** | | **Test for difference** | |
| --- | --- | --- | --- | --- | --- | --- | --- |
|  |  | <65 | >=65 | <65 | >=65 | I^2^, % | P_heterogeneity_ |
| Overall | 20 | 7162 | 5918 | 0.75 (0.69-0.81) | 0.79 (0.74-0.85) | <0.01 | 0.270 |
| Line of therapy |  |  |  |  |  |  |  |
| First line | 14 | 4736 | 4062 | 0.76 (0.69-0.84) | 0.80 (0.74-0.87) | <0.01 | 0.362 |
| Subsequent lines | 6 | 2426 | 1856 | 0.71 (0.62-0.83) | 0.76 (0.68-0.86) | 12.95 | 0.564 |
| Agent of immunotherapy |  |  |  |  |  |  |  |
| PD-1 inhibitor | 9 | 3180 | 2640 | 0.68 (0.57-0.81) | 0.76 (0.68-0.84) | 2.31 | 0.245 |
| PD-L1 inhibitor | 9 | 3248 | 2685 | 0.81 (0.74-0.88) | 0.80 (0.73-0.88) | <0.01 | 0.981 |
| Intervention |  |  |  |  |  |  |  |
| Immunotherapy alone | 12 | 4210 | 3409 | 0.76 (0.68-0.84) | 0.77 (0.70-0.85) | <0.01 | 0.890 |
| Chemo-immunotherapy | 8 | 2952 | 2509 | 0.73 (0.64-0.83) | 0.81 (0.73-0.90) | <0.01 | 0.130 |

IO: immunotherapy; NSCLC: non-small cell lung cancer; PD-1: programmed cell death 1; PD-L1: programmed cell death ligand 1; HR: hazard ratio; CI: confidence interval.

**Supplementary Table 3.** Differences in efficacy of IO vs control therapies in NSCLC patients by EGOG PS.

| **Variable** | **Studies No.** | **Patients No.** | | **Pooled HR (95% CI)** | | **Test for difference** | |
| --- | --- | --- | --- | --- | --- | --- | --- |
|  |  | ECOG=0 | ECOG=1 | ECOG=0 | ECOG=1 | I^2^, % | P_heterogeneity_ |
| Overall | 20 | 4583 | 8563 | 0.76 (0.69-0.83) | 0.76 (0.71-0.82) | <0.01 | 0.442 |
| Line of therapy |  |  |  |  |  |  |  |
| First line | 15 | 3351 | 6164 | 0.76 (0.68-0.85) | 0.78 (0.72-0.84) | <0.01 | 0.574 |
| Subsequent lines | 5 | 1232 | 2399 | 0.75 (0.65-0.86) | 0.74 (0.62-0.88) | 30.57 | 0.789 |
| Agent of immunotherapy |  |  |  |  |  |  |  |
| PD-1 inhibitor | 10 | 1993 | 4279 | 0.72 (0.63-0.83) | 0.69 (0.61-0.78) | <0.01 | 0.707 |
| PD-L1 inhibitor | 8 | 2106 | 3307 | 0.79 (0.71-0.88) | 0.83 (0.77-0.90) | 3.09 | 0.436 |
| Intervention |  |  |  |  |  |  |  |
| Immunotherapy alone | 11 | 2300 | 4688 | 0.76 (0.68-0.85) | 0.76 (0.68-0.86) | 4.20 | 0.709 |
| Chemo-immunotherapy | 9 | 2283 | 3875 | 0.75 (0.65-0.87) | 0.77 (0.70-0.85) | <0.01 | 0.704 |

IO: immunotherapy; NSCLC: non-small cell lung cancer; PD-1: programmed cell death 1; PD-L1: programmed cell death ligand 1; HR: hazard ratio; CI: confidence interval; ECOG PS: Eastern Cooperative Oncology Group performance status.

**Supplementary Table 4.** Differences in efficacy of IO vs control therapies in NSCLC patients by histology type.

| **Variable** | **Studies No.** | **Patients No.** | | **Pooled HR (95% CI)** | | **Test for difference** | |
| --- | --- | --- | --- | --- | --- | --- | --- |
|  |  | Squamous | Nonsquamous | Squamous | Nonsquamous | I^2^, % | P_heterogeneity_ |
| Overall | 12 | 2618 | 5364 | 0.74 (0.67-0.81) | 0.78 (0.70-0.87) | 14.91 | 0.396 |
| Line of therapy |  |  |  |  |  |  |  |
| First line | 7 | 1369 | 2710 | 0.72 (0.62-0.82) | 0.79 (0.67-0.93) | <0.01 | 0.175 |
| Subsequent lines | 5 | 1249 | 2654 | 0.76 (0.66-0.87) | 0.77 (0.67-0.88) | 35.63 | 0.956 |
| Agent of immunotherapy |  |  |  |  |  |  |  |
| PD-1 inhibitor | 6 | 1410 | 2856 | 0.72 (0.63-0.82) | 0.82 (0.68-0.97) | 28.80 | 0.207 |
| PD-L1 inhibitor | 5 | 981 | 2016 | 0.79 (0.68-0.92) | 0.76 (0.66-0.88) | 4.25 | 0.680 |
| Intervention |  |  |  |  |  |  |  |
| Immunotherapy alone | 11 | 2391 | 4872 | 0.75 (0.68-0.83) | 0.79 (0.71-0.88) | 22.28 | 0.485 |

IO: immunotherapy; NSCLC: non-small cell lung cancer; PD-1: programmed cell death 1; PD-L1: programmed cell death ligand 1; HR: hazard ratio; CI: confidence interval; ECOG PS: Eastern Cooperative Oncology Group performance status.

**Supplementary Table 5.** Differences in efficacy of IO vs control therapies in NSCLC patients by smoking history.

| **Variable** | **Studies No.** | **Patients No.** | | **Pooled HR (95% CI)** | | **Test for difference** | |
| --- | --- | --- | --- | --- | --- | --- | --- |
|  |  | Never | Former/current | Never | Former/current | I^2^, % | P_heterogeneity_ |
| Overall | 17 | 1831 | 9259 | 0.82 (0.70-0.96) | 0.76 (0.71-0.81) | 31.00 | 0.313 |
| Line of therapy |  |  |  |  |  |  |  |
| First line | 12 | 1167 | 5857 | 0.81 (0.66-0.99) | 0.76 (0.69-0.84) | 34.06 | 0.578 |
| Subsequent lines | 5 | 664 | 3402 | 0.83 (0.62-1.13) | 0.74 (0.68-0.81) | 36.67 | 0.380 |
| Agent of immunotherapy |  |  |  |  |  |  |  |
| PD-1 inhibitor | 7 | 885 | 3398 | 0.76 (0.56-1.03) | 0.70 (0.61-0.80) | 42.45 | 0.542 |
| PD-L1 inhibitor | 9 | 848 | 5240 | 0.83 (0.68-1.02) | 0.81 (0.76-0.87) | 0.79 | 15.401 |
| Intervention |  |  |  |  |  |  |  |
| Immunotherapy alone | 10 | 1098 | 5119 | 0.89 (0.72-1.09) | 0.75 (0.70-0.82) | 20.89 | 0.120 |
| Chemo-immunotherapy | 7 | 733 | 4140 | 0.74 (0.57-0.95) | 0.75 (0.67-0.85) | 40.46 | 0.938 |

IO: immunotherapy; NSCLC: non-small cell lung cancer; PD-1: programmed cell death 1; PD-L1: programmed cell death ligand 1; HR: hazard ratio; CI: confidence interval; ECOG PS: Eastern Cooperative Oncology Group performance status.
